# Supplementary material for: Modulating viscosity improves lentiviral transduction of NK cells: A simple solution to a persistent problem
Source: Mol Ther Adv. 2026 Feb 10;34(1):201692. doi: 10.1016/j.omta.2026.201692 (PMC13148914; doi:10.1016/j.omta.2026.201692)
Supplement: Document S1. Figures S1–S6 and Table S1 [file mmc1.pdf]

OMTA, Volume *34*

Supplemental information

Modulating viscosity improves lentiviral transduction of NK cells: A simple solution to a persistent problem

Mila Bjelica, Aissa Benyoucef, Hugo Romero, Kathie Beland, Etienne Gagnon, and Elie Haddad

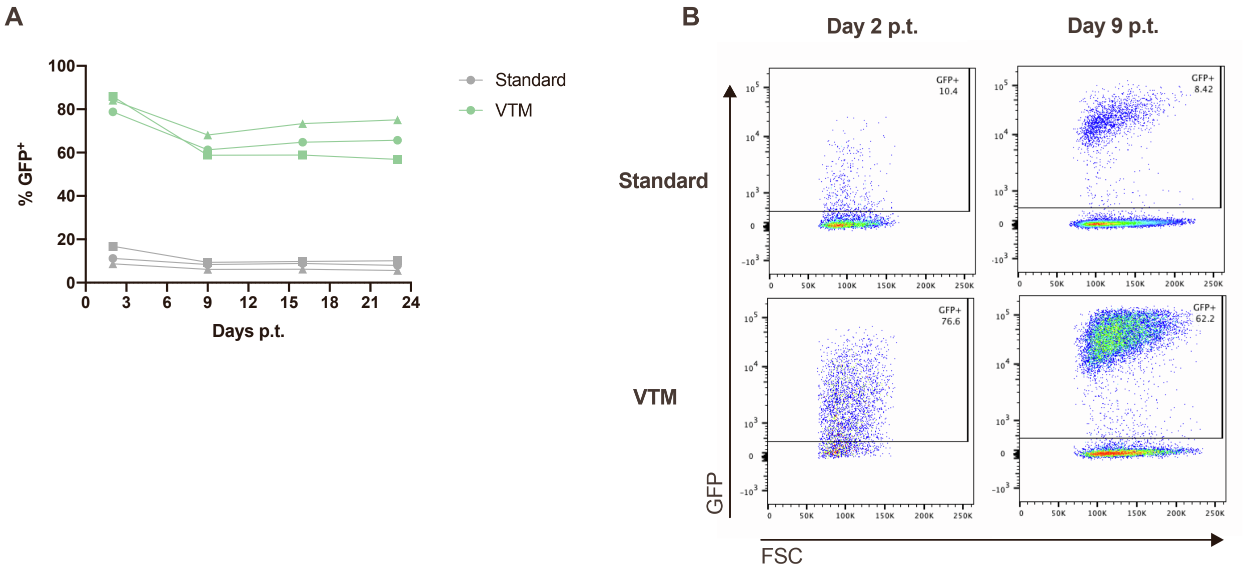

Figure S1. GFP over time A) GFP expression in NK cells post transduction (p.t.) with BaEV lentivirus, as NK are expanded and kept in culture. B) Representative flow cytometry plots at Day 2 and Day 9 post transduction. *n*=3 biological replicates.

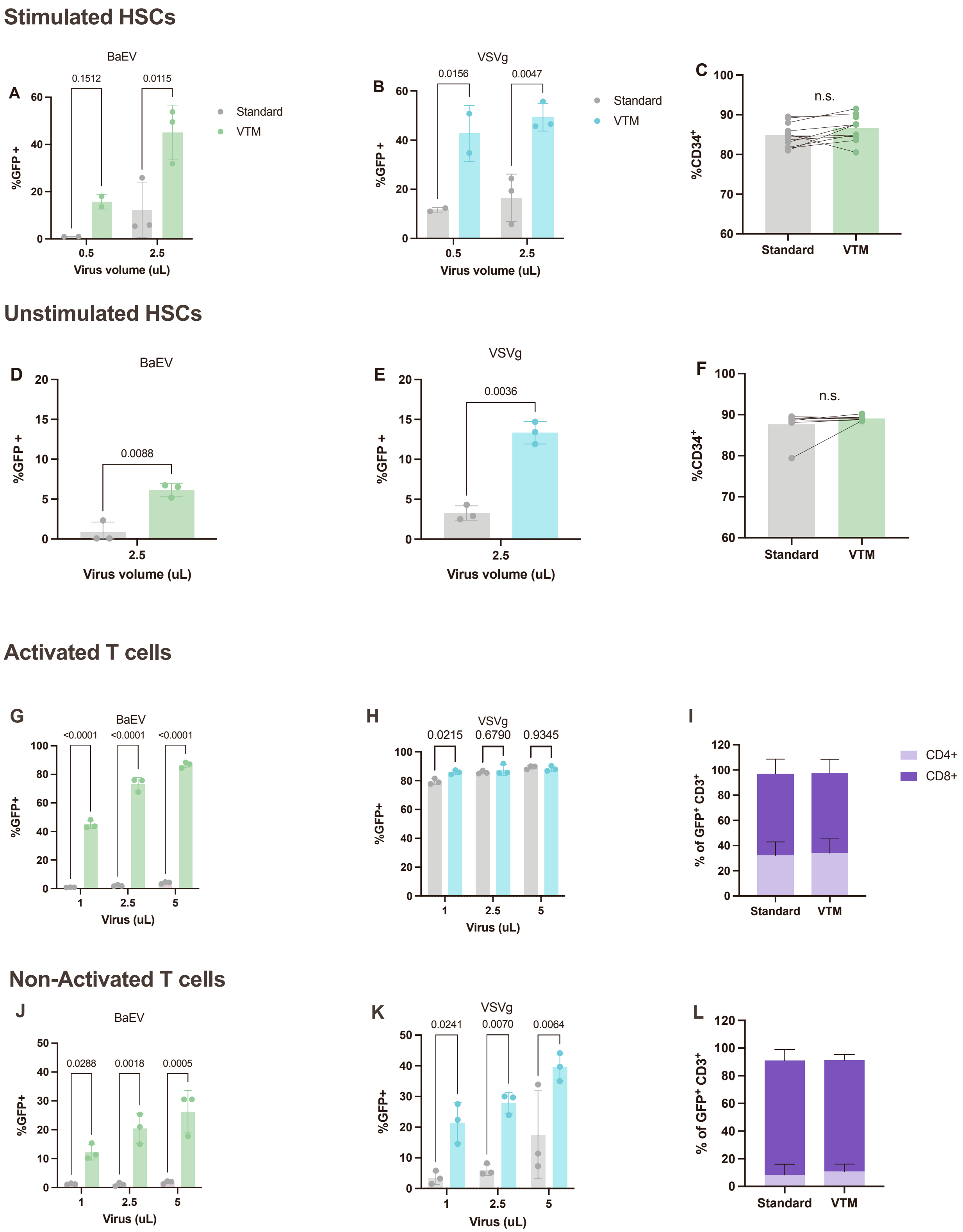

Figure S2. Transduction of HSCs and T cells in standard medium or VTM. (A-C) HSC were put in expansion medium overnight as stimulation prior to transduction, then expanded for 6 more days. A) Transduction efficiency HSCs with BaEV lentivirus at day

6 post transduction. B) Transduction efficiency of HSC with VSVg lentivirus at day 6 post transduction. C) %CD34+ of all samples pooled. (D-F) HSC were isolated and directly transduced then put in expansion for 6 days. D) Transduction efficiency of unstimulated HSC with BaEV lentivirus at day 6 post transduction. E) Transduction efficiency of unstimulated HSC with VSVg lentivirus at day 6 post transduction. F) %CD34+ of all samples pooled (G-I) T cells isolated and activated overnight prior to transduction, then expanded for 3 days. G) Transduction efficiency of T cells with BaEV lentivirus at day 3 post transduction. H) Transduction efficiency of T cells with VSVg lentivirus at day 3 post transduction. I) CD4 and CD8 populations in GFP+ CD3+ T cells all pooled. (J-L) T cells isolated and rested in RPMI for 3 hours prior to transduction, then kept in culture for 3 days. J) Transduction efficiency of T cells with BaEV lentivirus at day 3 post transduction. K) Transduction efficiency of T cells with VSVg lentivirus at day 3 post transduction. L) CD4 and CD8 populations in GFP+ CD3+ T cells all pooled. Significance was determined using a mixed-effects analysis with Sidak's multiple comparison test with a single pooled variance for experiments with multiple viral volumes or a paired single tail t test for experiments with a single viral volume. P values are reported above each comparison.

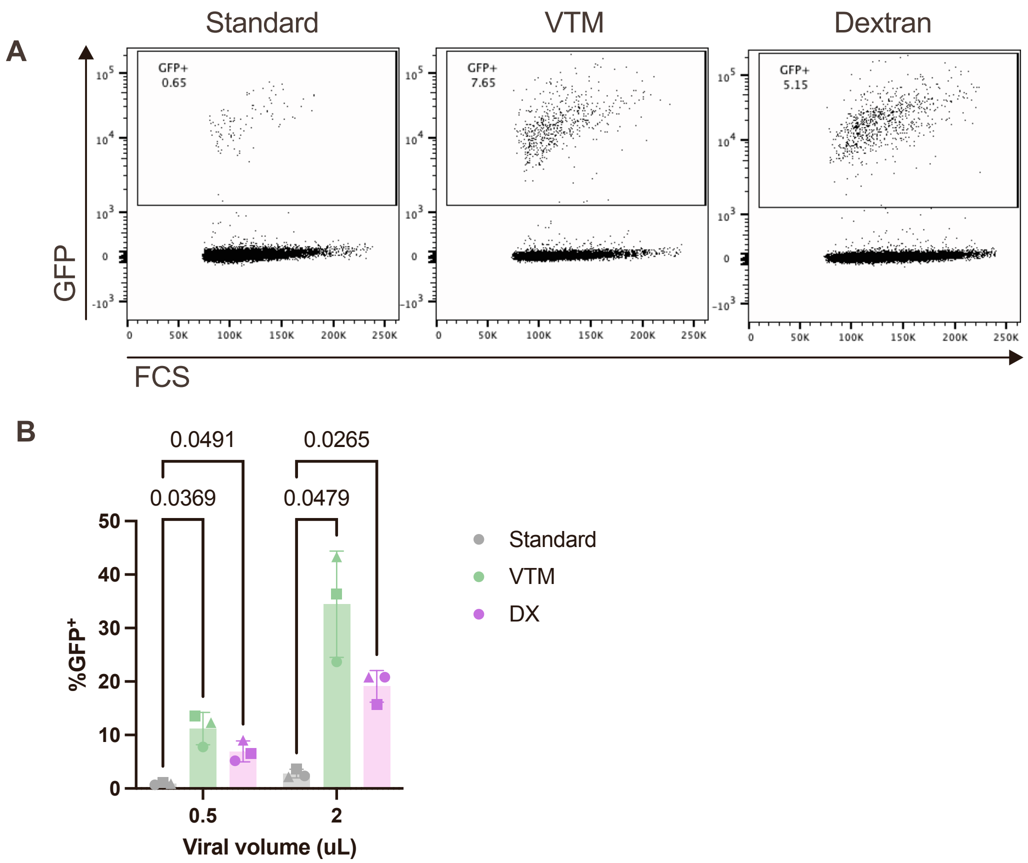

Figure S3. Dextran as an alternative thickening agent. A) Representative flow cytometry plots at Day 9 post transduction. B) Transduction efficiency of NK using BaEV lentivirus and standard medium, VTM or 3cps dextran (DX) supplemented medium at Day 9 post transduction. *n*=3 biological replicates. P values are reported above each comparison.

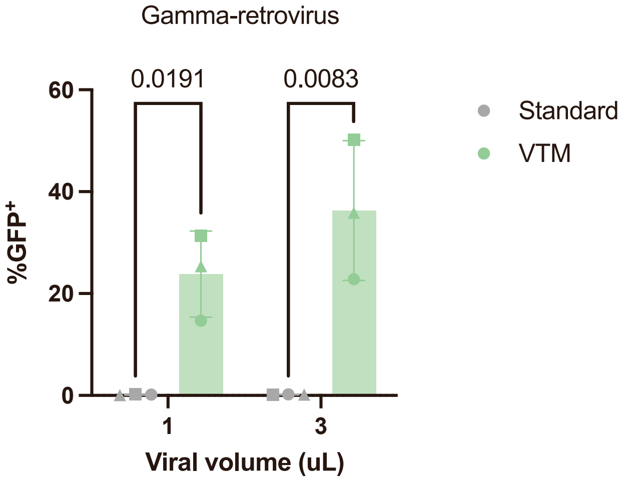

Figure S4. Transduction of NK cells with gamma-retrovirus in standard medium or VTM. A) Transduction efficiency with gamma-retrovirus at day 9 post transduction. *n*=3 biological replicates. P values are reported above each comparison.

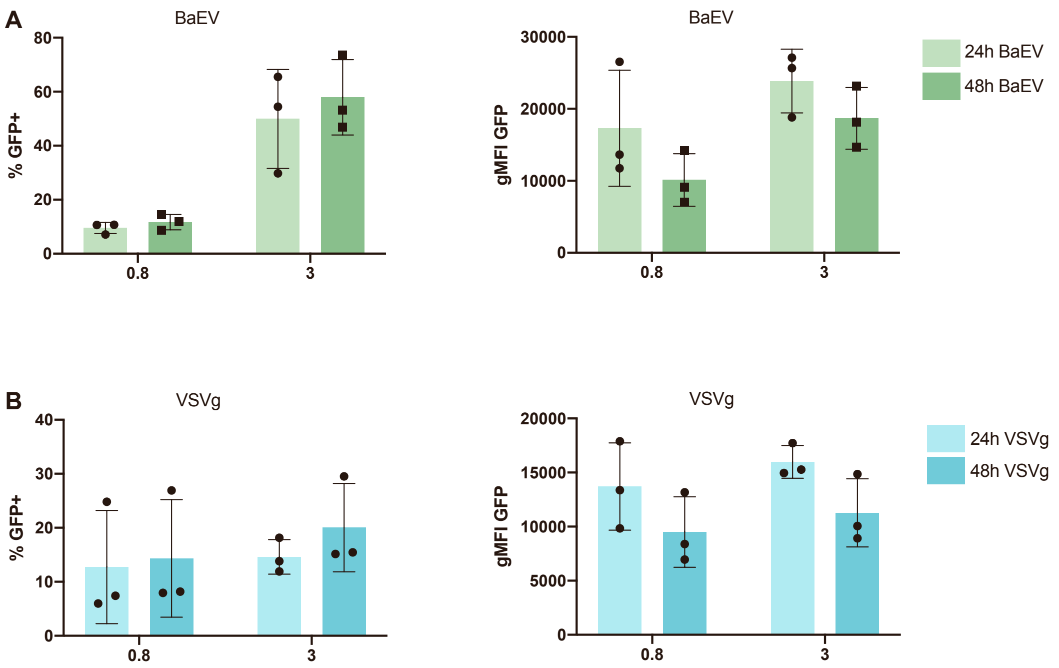

Figure S5. Transduction of NK using a 24 or 48 hour incubation with virus. A) Transduction efficiency with BaEV pseudotyped lentivirus at day 9 post transduction. B) Transduction efficiency with VSVg pseudotyped lentivirus at day 9 post transduction. *n*=3 biological replicates.

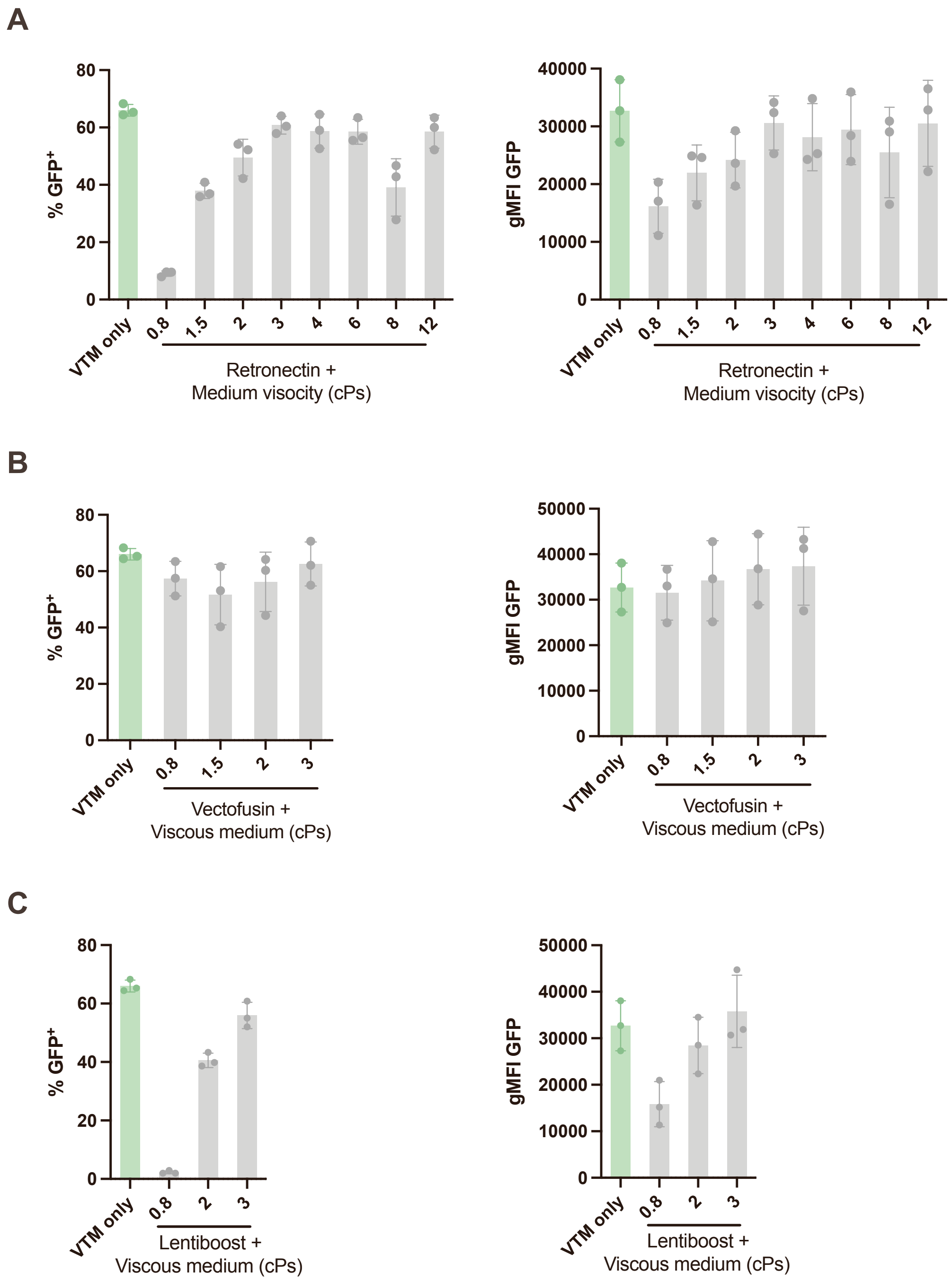

Figure S6. Additive effects of combining commercial transduction enhancers with viscous medium. Transduction efficiency and MFI of NK cells transduced with BaEV GFP in only 3cPs VTM or VTM of varying viscosity in combination with A) Retronectin B) Vectofusin C) Lentiboost. *n*=3 biological replicates. P values are reported above each comparison.

Table S1. Medium viscosity with corresponding methyl-cellulose concentrations. Adapted from Ma et al.[14]

**Medium viscosity (cPs)**	**Methyl-cellulose (w/v%)**
1.0	0.110
1.5	0.210
2.0	0.281
3.0	0.382
4.0	0.453
6.0	0.554
8.0	0.625
12.0	0.725
15.0	0.781
