# Supplementary material for: Modulating viscosity improves lentiviral transduction of NK cells: A simple solution to a persistent problem
Source: Mol Ther Adv. 2026 Feb 10;34(1):201692. doi: 10.1016/j.omta.2026.201692 (PMC13148914; doi:10.1016/j.omta.2026.201692)
Supplement: Document S2. Article plus supplemental information [file mmc2.pdf]

Molecular Therapy
Advances
Original Article

Modulating viscosity improves lentiviral transduction of NK cells: A simple solution to a persistent problem

Mila Bjelica,[1,2] Aissa Benyoucef,[2] Hugo Romero,[2] Kathie Beland,[2] Etienne Gagnon,[1,3] and Elie Haddad[1,2,4]

[1]Department of Microbiology, Infectiology and Immunology, Faculty of Medicine, University of Montréal, Montréal, QC, Canada; [2]CHU Sainte-Justine Azrieli Research Center, Montreal, QC, Canada; [3]Institut de Recherche en Immunologie et Cancérologie (IRIC), Montréal, QC, Canada; [4]Department of Pediatrics, Faculty of Medicine, University of Montréal, Montréal, QC, Canada

Genetically modified cell therapies (GMCT) hold potential for the treatment of cancer, autoimmune conditions, and rare diseases. A major determinant of GMCT success is modification efficiency, yet transducing certain cells of interest, such as primary natural killer (NK) cells, remains challenging. Major barriers to widespread adoption of GMCT are cost and difficulty of scale. Therefore, improving transduction rates with accessible, good manufacturing practices (GMP) compatible reagents may help reduce this barrier and prevent manufacturing failure. We show that using a modified viscous transduction medium (VTM) made with methyl-cellulose significantly increased the transduction efficiency of primary human NK cells without affecting viability, expansion, or function. VTM outperformed commercially available transduction enhancers. Transduction with VTM significantly improved the yield of anti-CD22 chimeric antigen receptor (CAR)-NK cells. Moreover, VTM was similarly effective at improving the transduction of primary T cells and hematopoietic stem cells. Overall, VTM may provide a simple, cost-effective, and GMP-compliant solution to increase the yield of genetically modified immune cells for immunotherapy. This may improve the efficacy of immunotherapies and reduce the overall costs of production.

INTRODUCTION

Genetically modified cell therapies (GMCT) are well-tested in treating cancers and are emerging as a promising treatment for autoimmune conditions and rare diseases.[1–4] For instance, the modification of T cells and natural killer (NK) cells with chimeric antigen receptors (CARs) has shown groundbreaking clinical benefits for hematological cancers.[1,2]

All FDA-approved CAR products are currently manufactured using viral vectors.[5] Among these, self-inactivating lentiviral vectors are generally favored over γ-retroviral vectors for their broader tropism and safer integration profile. While safe and effective, production queues for clinical-grade viral vectors are long and expensive. Therefore, it is highly desirable to optimize manufacturing processes to yield high-quality batches of good manufacturing practices (GMP) grade virus, while also optimizing transduction protocols to ensure that a single viral production can be used to produce as many batches of GMCT as possible.[6]

A major determinant of GMCT success is modification efficiency, since insufficient transduction may lead to therapeutic failure.[5] Obtaining consistently high transduction rates for immune cells still represents a notable technical challenge during manufacturing. This is especially true for primary human NK cells, which are notoriously difficult to transduce, perhaps due to their innate anti-viral capabilities and poor expression of low-density lipoprotein receptor (LDL-R), the receptor for classic vesicular stomatitis virus G protein (VSV-G) lentiviruses.[7] As such, efforts to improve NK cell transduction efficiency are ongoing. Previous studies have shown significant enhancements in NK cell transduction by pseudotyping vectors with envelope proteins that more readily bind NK surface receptors, such as baboon endogenous virus (BaEV), koala retrovirus (KoRV), or RD114 feline type C virus (RDF).[7–10] In parallel, studies using VSV-G-pseudotyped lentiviruses have shown improvements in NK cell transduction by pharmaceutical upregulation of the LDL-R receptor or inhibition of TLR inflammatory signaling pathways.[11,12] Other studies have focused on optimizing NK cell culture conditions and activation status prior to transduction.[13] While commercially available transduction enhancers have also been explored, they are often costly and not always effective.[5,10,12] Undeniably, major factors hampering more widespread adoption of GMCT are cost and scalability.[5] Improving transduction rates with accessible, GMP-compatible products may help reduce this barrier. There is, therefore, a clear need for an easy and accessible method to improve NK cell transduction.

Recently, Ma et al.. reported that increased extracellular fluid viscosity can improve transduction and transfection of various cell types by increasing vector uptake and endosomal escape.[14] Adopting this

Received 14 August 2025; accepted 6 February 2026;
https://doi.org/10.1016/j.omta.2026.201692.

Correspondence: Elie Haddad, Department of Pediatrics, Faculty of Medicine, University of Montréal, Montréal, QC, Canada.
E-mail: elie.haddad@umontreal.ca

© 2026 The Authors.
Published by Elsevier Inc. on behalf of The American Society of Gene and Cell Therapy.
This is an open access article under the CC BY license (http://creativecommons.org/licenses/by/4.0/).

approach to GMCT could not only improve the overall yield of genetically modified cells but may also provide a cost-effective solution for large-scale biomanufacturing. We ventured to optimize a viscous transduction medium (VTM) to improve the production of CAR-NK cells and assess the effects of this method on NK viability and function. In this study, we show that VTM offers a simple, accessible method to significantly improve primary human NK cell transduction rates, and lower the volume of viral vector needed to produce CAR-NK therapies without affecting NK viability, proliferation, or function.

RESULTS

Viscous medium significantly improves transduction of primary NK cells

Primary human NK cells are largely resistant to transduction by VSV-G lentiviruses.[7,8] While pseudotyping lentiviruses with baboon envelope proteins (BaEV) improves NK susceptibility to transduction, this strategy significantly reduces viral yield, making it challenging to achieve efficient transduction rates.[15,16] Titers were highly cell-type dependent, with HEK293T cells being very permissive to VSV-G but not to BaEV, while NK cells showed the inverse. (Figure 1A). We assessed the effect of transduction medium viscosity on primary human NK cell transduction using a VSV-G- or BaEV-pseudotyped lentivirus containing eGFP, driven by the EF1a promoter. NK cells were expanded with 4-1BBL mbIL-21 feeders and IL-2, then transduced using a consistent viral volume (1.7 uL) in standard medium (0.8 cPs) or in viscous medium (1.5–15 cPs). All transduction rates were reported after 1 week of expansion (9 days post-transduction) to avoid reporting incidences of pseudo-transduction, as GFP expression is initially elevated then stabilizes after 1 week (Figure S1). Using VTM significantly improved primary NK transduction with BaEV-pseudotyped vectors (Figures 1B and 1C). Transduction efficiency peaked at 3 cPs, yielding a 7.8 ± 2.0 ($p = 0.0036$)-fold increase in %GFP$^+$ NK cells.

To explore the effects of viral vector dose with respect to the observed VTM-mediated improvement in transduction efficiency, medium viscosity was fixed at 3cPs, and NK cells were transduced with increasing volumes of virus (Figure 1D). Interestingly, medium viscosity had a more important impact on transduction rates than the volume of transfection agent. Transduction rates using 0.25 μL of virus with VTM were comparable to 5 uL of virus in standard medium. Quintupling viral volume from 1 to 5 uL only yielded a 2.07-fold improvement in transduction, while increasing medium viscosity from 0.8 cPs to 3 cPs led to a 5.02-fold increase in transduction efficiency with just 1 uL of virus (Figure 1D). There were no differences in the viability of NK cells treated with standard medium or VTM (Figure 1E).

In contrast, VSV-G lentivirus transduction did not significantly improve with VTM (Figure 1F), suggesting that VTM-mediated improvements in transduction efficiency remain dependent on receptor-mediated endocytosis pathways.[17,18] Indeed, while there were some benefits to using VTM at lower viral volumes, the maximum transduction rates were comparable between VTM and standard transduction conditions, indicating a saturation of transduction with both approaches (Figure 1G). Even so, non-activated and activated T cells, as well as unstimulated and stimulated CD34$^+$ hematopoietic stem cells (HSCs), were successfully transduced by both VSV-G and BaEV vectors, and transduction significantly improved with VTM (Figure S2). These differences between cell types may be due to the availability of viral entry receptors, as NK cells scarcely express the VSV-G receptor (LDL-R), but highly express the BaEV receptors (ASCT1/2).[7,8] Dextran was tested as an alternative thickening agent, and we observed that it also significantly increased transduction, albeit slightly less than VTM (Figure S3), thereby strongly suggesting that the effect is related to viscosity rather than another property of methyl-cellulose. Interestingly, VTM was also found to significantly improve NK cell transduction by gamma-retrovirus (Figure S4).

Finally, increasing transduction time from 24 to 48 h did not impact transduction efficiency for BaEV nor VSV-G vectors (Figure S5).

Treatment with VTM does not affect viability, cytotoxic function, or transcriptional profile of NK cells

To investigate the potential impact of VTM on primary NK cells, we incubated NK cells in 3 cPs VTM or standard medium (0.8 cPs) for 24 h, then washed and rested them in complete medium for 48 h. This mimicked the transduction protocol without adding virus. VTM did not affect NK cytotoxic capacity, as measured by a classical cytotoxic assay against K562, nor did it impact degranulation, as measured by CD107a surface expression, or cytokine production (Figures 2A and 2B). To better assess the potential impact of using VTM, we performed bulk RNA-seq analysis on samples collected at each stage to explore changes in transcriptional profiles (Figure 2C). Principal-component analysis (PCA) revealed that, upon recovery, profiles of VTM-treated and standard medium-treated NK cells clustered together for each donor upon resting (Figure 2D). Furthermore, there were no major differences in the top 100 differentially expressed genes between NK cells treated with VTM or standard medium. In fact, inter-donor variability was more evident than the effects of any treatment. (Figure 2E).

VTM outperforms most commercially available transduction enhancers

We compared the ability of 3 cPs VTM and popular commercial transduction enhancers to improve NK cell transduction efficiency with BaEV lentivirus. Commercial enhancers included LentiBoost (Revvity), ViralEntry (Applied Biological Materials), Vectofusin (Miltenyi Biotech), and RetroNectin (Takara). VTM was the most effective at improving transduction rates (Figures 3A and 3B). VTM provided the highest yield of transduced cells 9 days post-transduction (Figure 3C). Furthermore, there were no additive effects when VTM was combined with existing transduction enhancers, suggesting that VTM already maximizes transduction (Figures S6A–S6C).

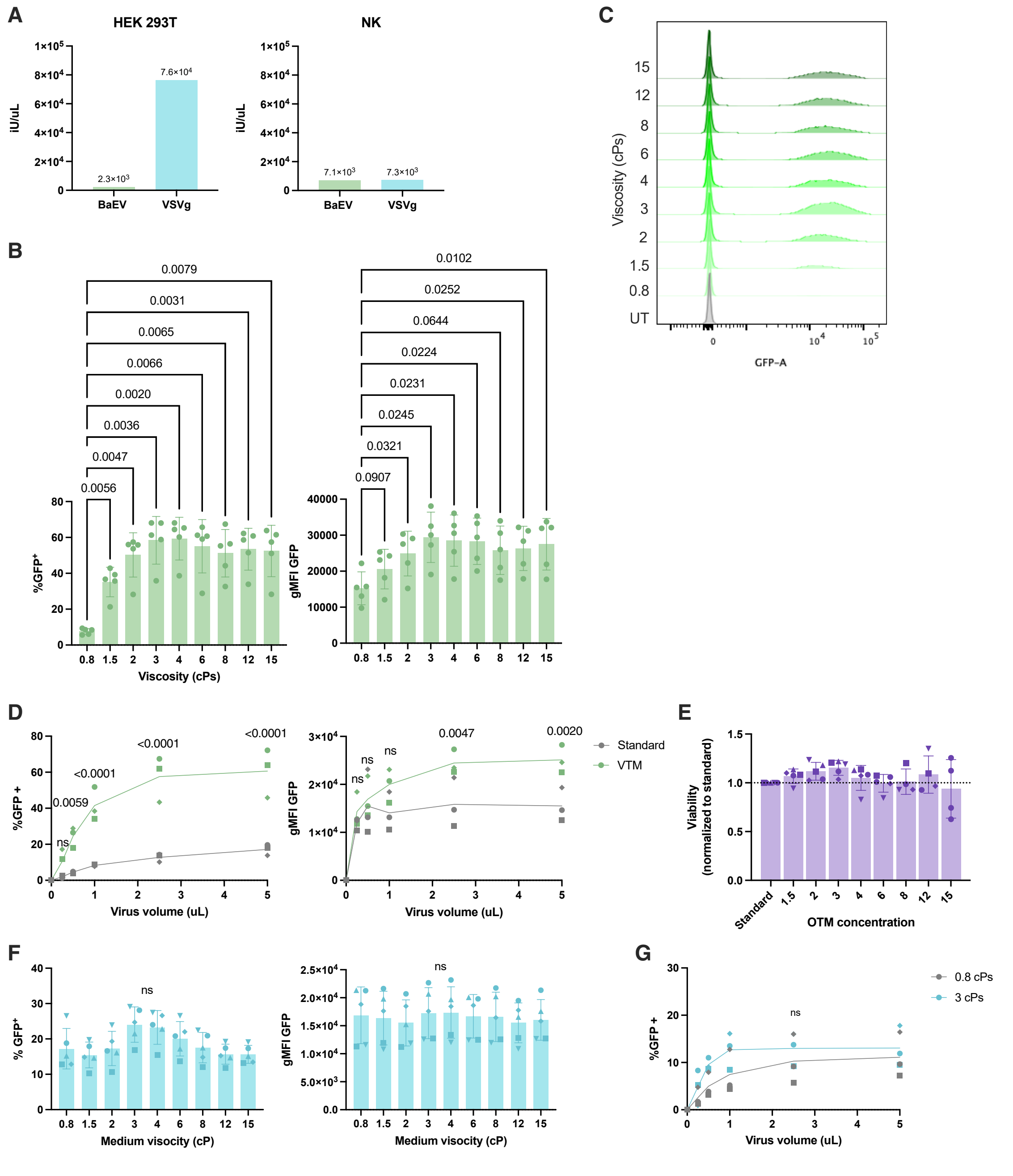

Figure 1. Effects of medium viscosity on lentiviral transduction efficiency

(A) Mean eGFP lentivirus titers of a single lentiviral production for BaEV and VSV-G as determined by functional titration on HEK293T cells or primary NK cells. (B) Transduction efficiency and geometric mean fluorescence intensity (gMFI) of 1×10^5 primary NK cells transduced with lentivirus in medium of increasing viscosities, reported 9 days post transduction (n = 5 biological replicates). (C) Representative flow cytometry histograms at 9 days post transduction. (D) Transduction efficiency and gMFI of 1×10^5 NK cells transduced with increasing amounts of BaEV virus in 3 cPs VTM (n = 5 biological replicates). (E) Viability of NK cells treated with VTM of varying viscosities. (F) Transduction efficiency and gMFI of primary NK cells transduced with VSV-G lentivirus in medium of increasing viscosities, reported 9 days post transduction (n = 3 biological replicates). (G) Transduction efficiency of 1×10^5 NK cells transduced with increasing amounts of VSV-G virus in 3 cPs VTM (n = 3 biological replicates). Statistical significance was determined using (B) and (F) an RM one-way ANOVA with Dunnett's multiple comparison test or (D) and (G) a mixed-effects analysis with Sidak's multiple comparison test. Data are represented as mean ± SD. p values are indicated above each comparison. ns, not significant.

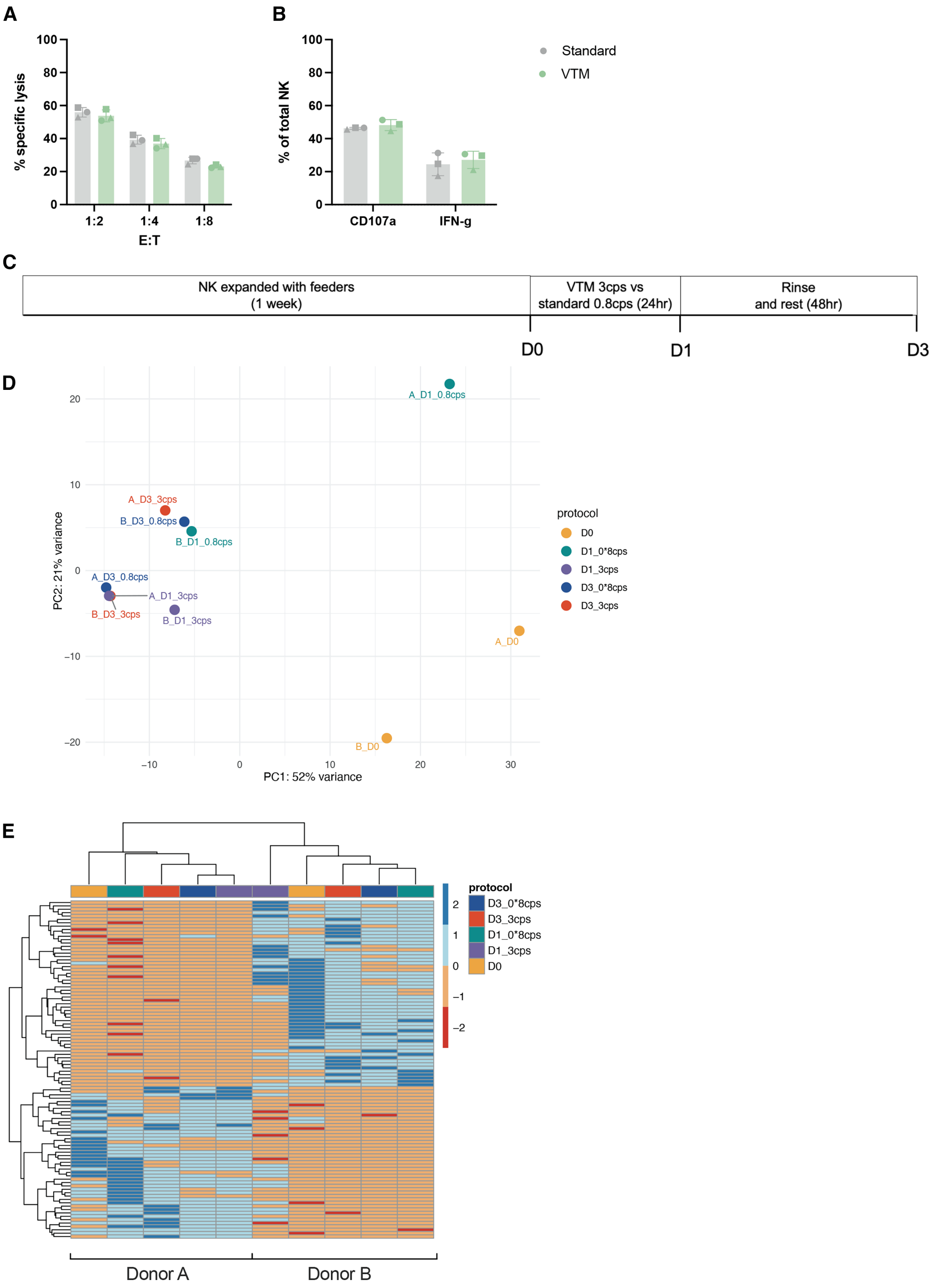

(legend on next page)

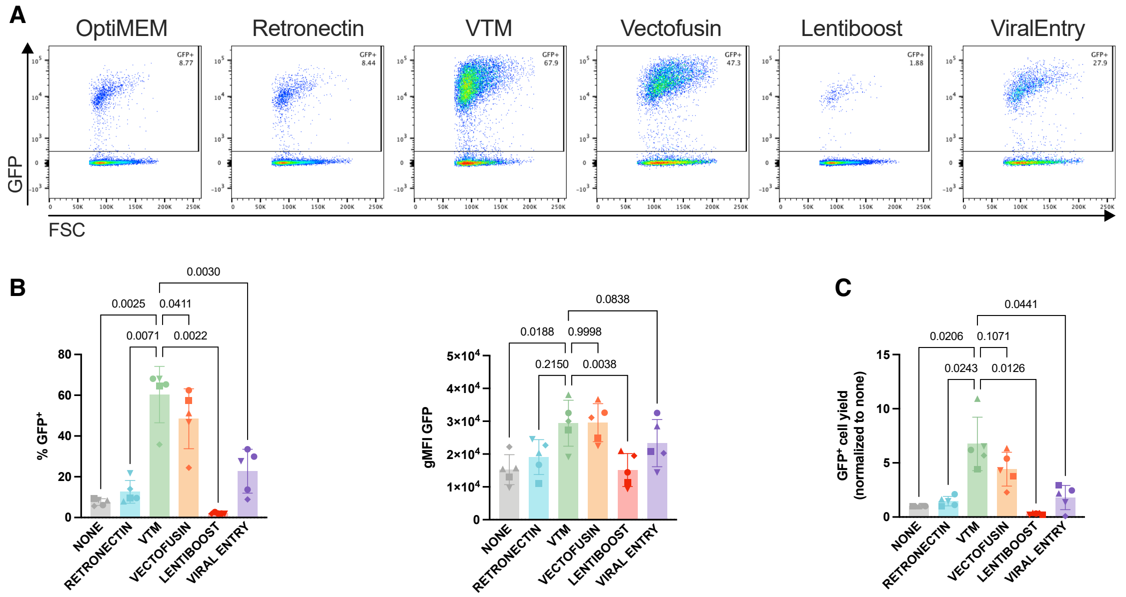

Figure 3. Effects of VTM or commercial transduction enhancers on NK transduction

(A) Representative flow cytometry plots of NK cells transduced with BaEV GFP using different transduction enhancers at 9 days post-transduction. (B) Comparison of NK cell transduction levels using different enhancers or 3 cPs VTM at 9 days post-transduction. (C) Modified NK cell yield 9 days post-transduction. $n = 5$ biological replicates. Statistical significance was determined using an RM one-way ANOVA with Greenhouse-Geisser correction and Dunnett's multiple comparisons test. Data are represented as mean ± SD. p values are reported above each comparison.

VTM improves CAR-NK transduction and facilitates scale-up

As a proof of concept for cell therapy production, we tested our transduction method using the well-established anti-CD22 CAR with a 2A blue fluorescent protein (BFP) reporter (Figure 4A).[19] BaEV viral titers for the CAR construct were lower than for eGFP, likely due to a larger transgene size (Figure 4B). When CAR surface expression was assessed by staining with Siglec-2 (CD22 cognate peptide), BFP and CAR were highly correlated (Figure 4C). Despite low viral titers, transduction with VTM increased CAR expression by 4.66 ± 1.66-fold ($p < 0.0001$) (Figure 4D). As expected, NK cells with higher transduction levels and CAR expression showed more killing of CD22$^+$ B-ALL target cells (RS4;11) (Figure 4E). VTM provided a more potent product due to the increase in the proportion of transduced CAR-NK cells capable of targeting cancer cells.

VTM significantly improves the transduction of a large Cas9 transgene

Lentiviral packaging is typically limited to ∼10 kb, with packaging efficiency decreasing as plasmid size increases.[20] Given this constraint, strategies to enhance transduction efficiency of large transgenes are essential. We opted to show a second application for VTM with NK gene knockout, using a large Cas9 transgene. Primary human NK cells were transduced with a lentiviral vector encoding Cas9-BFP and a guide RNA targeting the *AAVS1* locus, with a total construct size of ∼9.5 kb (Figure 5A). VTM significantly improved transduction of Cas9-BFP compared to standard medium (Figure 5B). To assess Cas9 activity, transduced cells were sorted into BFP$^+$ and BFP$^-$ populations, and genome editing was evaluated using a T7 endonuclease I (T7E1) assay. PCR amplicons (∼500 bp) spanning the targeted *AAVS1* locus were generated (Figure 5C). The presence of cleavage products in BFP$^+$ cells (Figure 5D) and the lack of cutting in the BFP$^-$ group confirmed active Cas9-mediated genome editing.

DISCUSSION

GMCT have shown great potential in treating a wide range of diseases.[1–4] More specifically, NK cells offer an interesting platform for GMCT due to their capacity for allogenic transfer and high safety profile, characterized by a low risk of cytokine release syndrome and no risk of graft-versus-host disease.[21] NK cells also express a variety of innate activating and inhibitory receptors to help detect and destroy malignant cells and participate in antibody-dependent cellular cytotoxicity (ADCC), while sparing healthy tissue.[21] Genetic

Figure 2. Effects of VTM treatment on NK viability, cytotoxic function, and transcriptional profile

(A and B) (A) Target cell lysis and B) NK cell activation and IFN-γ expression following a 3-h incubation with K562 target cells ($n = 3$ biological replicates). Data are represented as mean ± SD. (C) Schematic of experimental conditions for RNA-seq. (D) PCA plot of NK cells pre- and post-incubation in 3 cPs VTM or standard medium. (E) Top 100 differentially expressed genes clustered by similarity. Donors are denoted as (A) or (B) ($n = 2$ biological replicates). Statistical significance was determined using mixed-effects analysis with Sidak's multiple comparison test or a paired, two tailed t test. ns, not significant.

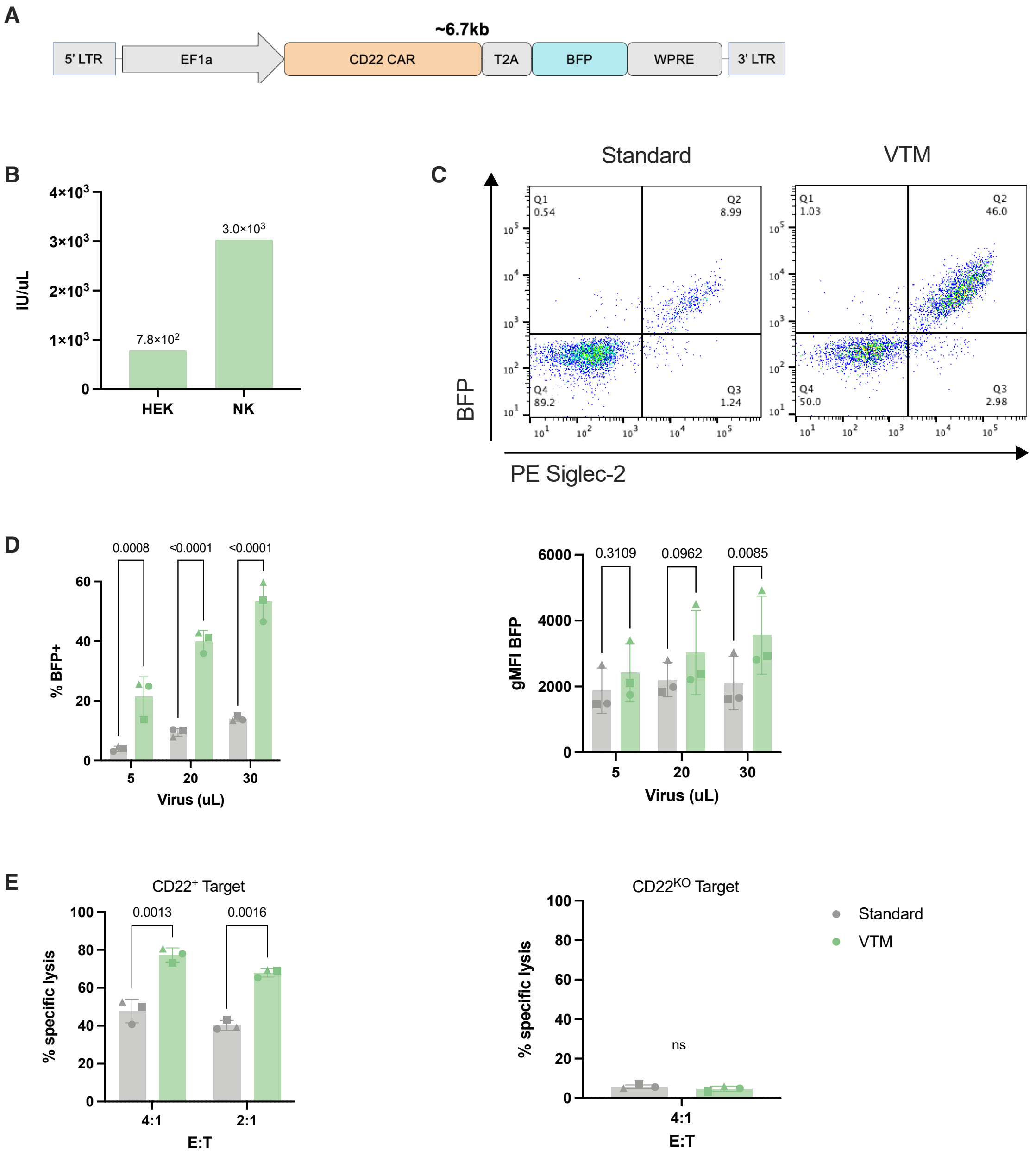

Figure 4. Transduction of anti-CD22 CAR into primary NK cells using VTM

(A) Schematic representation of the transgene, with approximate cargo size between long terminal repeats (LTRs). (B) Functional viral titers determined using HEK293T cells or primary NK cells. (C) Flow cytometry plots showing BFP expression against CAR CD22 surface expression assessed with Siglec-2. (D) Transduction efficiency of NK cells 9 days post-transduction with anti-CD22 CAR BFP. (E) Cytotoxic assay using the 20 uL condition against RS4;11 cells expressing CD22 and RS4;11 CD22 KO. Statistical significance was determined using a mixed-effects analysis with Sidak's multiple comparison test for experiments with multiple viral volumes or E:T ratios. A paired, two-tail *t* test was used for experiments with a single E:T ratio. Data are represented as mean ± SD. *p* values are reported above each comparison. ns, not significant.

modification can be used to boost these intrinsic anti-cancer capabilities by improving target recognition, cell metabolism, persistence, homing, and more. Unfortunately, NK cells remain difficult to transduce.

Several studies have tried to improve NK transduction levels by modifying the lentiviral envelope protein, optimizing NK culture conditions, and using various transduction enhancers.[7,8,10–13] Indeed, NK cells are poorly transduced by classic VSV-G-pseudotyped lentiviruses

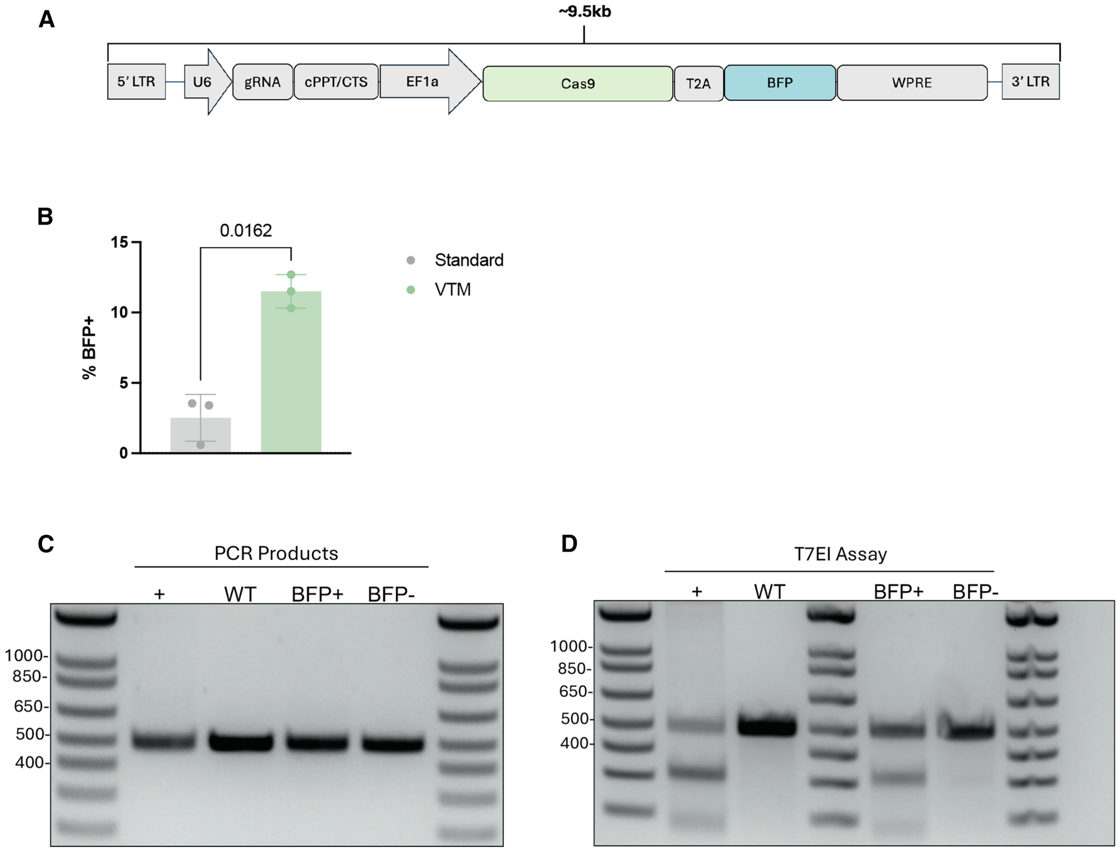

Figure 5. Transduction of Cas9 into primary NK cells using VTM

(A) Schematic representation of the transgene, with approximate cargo size between LTRs.

(B) Transduction efficiency of NK cells 9 days post-transduction with Cas9-BFP ($n = 3$ biological replicates). Statistical significance was determined using a paired, two-tailed *t* test. Data are represented as mean ± SD. *p* values are reported above each comparison. (C) PCR amplicons of the targeted *AAVS1* region, including an electroporated Cas9 + *AAVS1* single guide (sg)RNA + homology directed repair (HDR) template positive control (+), wild type (WT) NK control, and sorted BFP+ and BFP- NK cells from the VTM condition. (D) Representative gel image of the T7E1 assay, including an electroporated Cas9 + *AAVS1* sgRNA + HDR template positive control (+), WT NK control, and sorted BFP+ and BFP- NK cells from the VTM condition.

due to scarce expression of LDL-R (the VSV-G receptor).[7] Pseudotyping vectors with BaEV improves NK cell transduction due to their abundant expression of ASCT1/2[7,8]; however, this strategy reduces viral titers, especially for larger expression cassettes.[15] This, in turn, represents a significant issue for NK-GMCT production and scale-up for clinical use. We show herein that using VTM can effectively improve transduction, requiring less vector and leading to a higher yield of modified NK cells, even with poor viral titers. Combining VTM with strategies to improve BaEV viral titers could further improve the production of genetically modified NK cells.[15,16,22]

Previous work in HEK293T cells has suggested that viscosity-mediated improvements in adeno-associated virus (AAV) transduction are heavily dependent on macropinocytosis and clathrin- and caveolae-independent processes.[14] Supporting this, viscous extracellular fluid can increase actin remodeling and motility in adherent cell lines,[23] which may promote or enhance macropinocytosis.[14,24] Macropinocytosis is a form of non-selective fluid phase endocytosis that may be growth factor-induced or constitutive in certain cell types.[25] Naturally, preferences for endocytic pathways are highly cell-type and cargo-specific.[17] In our study, VTM considerably improved NK cell transduction with BaEV, but not VSV-G vectors, in agreement with NK cell expression of the receptors targeted by these viral proteins. However, both activated and non-activated T cells, as well as stimulated and unstimulated HSCs, were amenable to VTM-mediated improvement in transduction of both VSV-G and BaEV vectors. This suggests that VTM-mediated enhancements of NK cell lentiviral transduction remain reliant on ligand-dependent endocytic pathways rather than non-specific macropinocytosis. Finally, the observation that transduction efficiencies could be saturated at low volumes of lentiviral vectors also points to a receptor-mediated endocytosis rather than fluid-phase pinocytosis.[26] Therefore, we hypothesize that, in our context, viscous medium boosts transduction by increasing the incidence and duration of receptor-ligand interactions. Hence, it could increase the transduction of

NK cells for BaEV, but not for VSV-G, since it could not overcome a lack of an entry receptor. While cognate ligand expression on cargo may contribute to increased bystander macropinocytosis,[27] macropinocytosis does not appear to be the primary mechanism driving transduction improvement by VTM in NK cells. Interestingly, dextran-thickened medium also significantly increased transduction, supporting the role of viscosity. However, VTM remained slightly more effective, not excluding an additional mechanism for methyl-cellulose. A prior study reported enhanced NK cell transduction using the cationic additive diethylaminoethyl (DEAE)-dextran hydrochloride, a positively charged dextran derivative, without exploring the effect of viscosity.[28]

Next, we showed that VTM outperformed common transduction enhancers. Commercially available transduction enhancers are proprietary and costly, especially when manufacturing at clinical scales. Moreover, certain enhancers, like retronectin, require ∼6.5 h from preparation of plates to viral coating and transduction. Methyl-cellulose is widely accessible, generally considered GMP-compatible, and easy to incorporate into standard workflow.[29] In turn, VTM has the potential to save time and resources for manufacturers. This may be particularly important in the context of non-commercialized cell therapies and academic laboratories.[30]

We applied VTM to CAR-NK production and showed improved yield of genetically modified cells. As expected, this higher proportion of modified cells translated into improved cytolytic function in *in vitro* tests. There is a possibility that such increases in CAR expression could be associated with better clinical outcomes. Transduction rates in clinical CAR-NK trials are highly variable, with some infusions containing as little as 23% CAR^+ NK cells but still achieving positive results.[1,31] Using VTM may enable more consistent transduction rates or allow for doses with lower cell volume but a higher proportion of modified cells.

A limitation of this study is that this strategy was not assessed in the context of clinical-grade production or with an automated cell therapy manufacturing platform. While slightly thicker than standard medium, VTM manipulation does not require syringes and remains easy to dispense with regular pipette tips. We believe that the option to customize flow pressure and medium washes in existing closed cell systems would allow for relatively simple incorporation. Additionally, we did not assess VTM-treated NK cells *in vivo*. While our work focused on BaEV-pseudotyped vectors for NK cells, we speculate that this strategy could be broadly applicable to difficult-to-transduce cell types when using compatible vectors, as supported by our preliminary data in unstimulated HSCs and non-activated T cells.

All in all, we show an easily adaptable method to improve the transduction of primary human NK cells using VTM, which primarily relies on improving viral ligand-cell receptor interactions. VTM offers a cost-effective solution to variable transduction efficiencies and allows manufacturers to maximize a single production lot of viral vector. We believe this strategy may facilitate the manufacturing of GMCT and scale-up, as well as minimize associated costs, especially for hard-to-transduce cell types such as NK cells. The addition of VTM to the transduction workflow is a simple modification that may enable a single viral preparation to generate 5-fold more GMCT at a lower cost than commercial transduction enhancers.

MATERIALS AND METHODS

Primary cells

Blood samples were obtained from consented, healthy, adult donors. All methods were approved by the CHU Sainte-Justine Research Ethics Board (CHUSJ REB approval 2024–6256 & 3195). Peripheral blood mononuclear cells (PBMCs) were isolated by density gradient centrifugation. NK cells were activated and expanded using irradiated K562 4-1BBL mbIL-21 feeder cells and IL-2 (100 U/mL) in R10: RPMI 1640 supplemented with 10% fetal bovine serum (FBS) and 1% penicillin/streptomycin (Gibco). Remaining CD3+ cells were depleted using the EasySepTM Human CD3 Positive Selection Kit II (StemCell Technologies, Vancouver BC, Canada). Cells were subsequently stimulated once a week with feeders and regularly maintained in R10 containing IL-2 (100 U/mL).

T cells were isolated from PBMCs using the EasySep Human CD3 Positive Selection Kit II (StemCell). Cells were maintained in complete medium with IL-7 and IL-15 (10 ng/mL).

Cord blood was obtained from HemaQuebec. HSCs were isolated from cord blood mononuclear cells using the CD34 Microbead UltraPure kit (Miltenyi Biotech, North Rhine-Westphalia, Germany). HSCs were cultured in expansion medium consisting of StemSpanII supplemented with GlutaGro(1×), penicillin/streptomycin (1×), stem cell factor (100 ng/mL), thrombopoietin (50 ng/mL), FLT3L (100 ng/mL), and UM729 (500 nM). Medium was replenished every 3 days, and cells were split when confluent.

Cell lines

K562 and RS4;11 cells were purchased from ATCC (Manassas, Virginia, USA). RS4;11 GFP and RS4;11 GFP $CD22^{KO}$ were generated as previously described (Colamartino). Cell lines were cultured in R10. K562 4-1BBL mbIL-21 feeder cells were kindly provided by Dean A. Lee,[32] grown in R10, and irradiated at 100 Gy prior to co-culture with NK cells.

Plasmids and viral vectors

A full human EF1a promoter sequence was cloned into the pHRSIN-SFFV-eGFP plasmid in place of the spleen focus-forming virus (SFFV) promoter.[33] For CAR vectors, the eGFP was replaced with an anti-CD22 CAR (m971 ScFv) joined to a CD28TM domain, a 4-1BBz co-stimulatory domain, and a CD3 zeta chain.[19] A P2A BFP was added to serve as a reporter protein. For the Cas9 plasmid, the tetR from pEJS614_pTetR-P2A-BFPnls/sgNS (Addgene plasmid # 108650)[34] was replaced with a Cas9 sequence. A guide sequence targeting AAVS1 (5′GGGGCCACTAGGGACAGGAT) was inserted

behind the T7 promoter. Self-inactivating HIV-based lentiviruses with a psPAX2 packaging system were produced as described previously by Colamartino et al.[8] Briefly, HEK293T (ATCC, Virginia, USA) packaging cells were transfected with the VSV-G or BaEV envelop plasmid, transfer plasmid, and packaging plasmid using polyethylenimine (PEI) (Sigma-Aldrich, Missouri, USA). For gamma-retrovirus, transfection was performed with pMKO.1 GFP (William Hahn, Addgene plasmid #10676), pBS-CMV-gagpol (Patrick Salmon, Addgene plasmid #35614), and the RD114 envelope plasmid (Jakob Reiser, Addgene plasmid #17576).[35] Viral supernatant was concentrated using LentiX (Takara, Shiga, Japan) as per the manufacturer's instructions. Virus was concentrated 200× in OptiMEM (Gibco) and stored in small aliquots at −80°C. Titration was performed on HEK293T cells and/or NK cells and acquired by flow cytometry after 72 h of incubation.

VTM

Methyl-cellulose stock solution (3%) in Iscove's Modified Dulbecco's Medium (IMDM) (56 kDa, R&D systems) was used as a thickening agent that does not significantly affect medium osmolarity.[23] Following the concentrations reported by Ma et al. (Table S1),[14] the methyl-cellulose solution was mixed with a suitable culture medium to obtain the desired viscosities.

Transduction

After 7 days of expansion with feeder cells, NK cells were plated at 10^5 cell/well in a 96-well plate with a defined amount of lentivirus and IL-2 (100 U/mL final concentration). This was supplemented with the appropriate viscosity medium to achieve the desired final viscosity. Lentiboost (Revvity, Massachusetts, USA), ViralEntry (Applied Biological Materials, British Columbia, Canada), and Vectofusin-1 (Miltenyi Biotech, North Rhine-Westphalia, Germany) were used at a dilution of 1:100, as per the manufacturer's instructions, in a total of 200 uL final volume. Flat-bottom, non-tissue-culture treated 96-well plates were coated with RetroNectin (Takara, Shiga, Japan) up to 4 days before transfection and kept sealed at 4°C until use. On the morning of transfection, viruses were added to RetroNectin-coated plates, and they were incubated for 4 h at 37°C before adding NK cells. After 24 h of incubation in the transduction medium, cells were washed with warm medium and resuspended in complete culture medium. The following day, cells were counted by flow cytometry and put into expansion.

Freshly isolated, non-activated T cells were rested in RPMI for 3 h prior to transduction. A portion of T cells was stimulated for 24 h with CD3/CD28 Dynabeads (Gibco) at a ratio of 3:1 in R10 supplemented with IL-7 and IL-15 (10 ng/mL). Cells were transduced at 10^5 cell/well in a 96-well plate with a defined amount of lentivirus and IL-7 and IL-15 (10 ng/mL final concentration), supplemented with VTM. After 24 h of incubation in the transduction medium, cells were washed with warm R10 and resuspended in culture medium.

HSCs were transduced in a stimulated or resting state. HSCs were stimulated in expansion medium for 1 day prior to transduction, as described above. Cells were transduced in culture medium without UM729, with or without VTM. Resting HSCs were transduced immediately after isolation in StemSpanII supplemented with rapamycin (5 nM final) and CHIR (3 uM final), with or without VTM. After 24 h of incubation in the transduction medium, cells were washed with warm OptiMEM and resuspended in expansion medium.

Assessment of genomic double-stranded breaks in *AAVS1*

Transduced cells were sorted into BFP^+ or BFP^- fractions and re-expanded to obtain sufficient sample for subsequent tests. Cells were harvested, and gDNA was isolated using a QIAGEN DNeasy Blood and Tissue Kit. The targeted *AAVS1* region was amplified by PCR using Platinum II Taq Hot-Start polymerase (Invitrogen) and primers 5′ TTCTCCTGTGGATTCGGGTCAC 3′ and 5′ CTC TCTGGCTCCATCGTAAGCA 3′, as validated and reported by Karaoudi et al.[36]

Amplicons were treated with T7E1 (New England Biolabs), as directed by the manufacturer. Samples were run on a 1.5% agarose gel with a 1kb+ ladder (Invitrogen).

Flow cytometry

Transgene expression (GFP and BFP) and cell counts were assessed by flow cytometry using CountBright counting beads (Thermo Fisher Scientific). For CAR surface expression, cells were stained with Siglec2 (CD22)-Fc chimeric peptide (R&D) and an anti-Fc-PE secondary antibody (Jackson ImmunoResearch). 7-amino-actinomycin D (7-AAD) was used to discriminate live cells. Cells-specific markers included BV421-CD34, CD3-FITC, CD3-PE, and CD56-BV421 (Biolegend).

Cytotoxic assays

To assess the effects of VTM on NK function, K562 cells were tagged with PKH26 (Sigma-Aldrich) and plated in a 96-well U-bottom plate to serve as target cells. For CAR-NK assays, RS4;11 GFP and RS4;11 GFP $CD22^{KO}$ served as target cells. NK cells were added at different effector-to-target ratios, except for a set of control wells reserved for targets only. Plates were incubated for 24 h at 37°C, 5% CO_2. Post incubation, 7-AAD was added to the wells, and the plate was acquired by flow cytometry. Specific lysis was calculated as follows:

$$\%\text{Specific Lysis} = \left(100 - \text{Live Targets}_{Sample} \middle/ \text{Live Targets}_{control}\right) \times 100$$

RNA seq

RNA was isolated using the RNEasy Plus kit (Qiagen, Hilden, Germany) and stored at −80°C until sequencing. For all samples,

RNA integrity number (RIN) exceeded 9.6. Sequencing libraries were cleaned using the NEB HMR rRNA depletion kit. Next-generation sequencing was performed by Genome Quebec (Montreal, Quebec) using the Illumina NovaSeq PE100 at a setting of 25 M ± 5 M reads.

Initial RNA-seq data was generated and processed at Genome Quebec CES (Montreal, QC, Canada). RNA-seq data has been deposited un BioProject: PRJNA1427148. Binary alignment map (BAM) files were sorted and indexed with Samtools, and gene-level read counts were obtained using the countOverlaps function (IRanges) with GENCODE GRCh38.p13 (gencode.v35) annotations. Raw count tables were then imported into R, ENSEMBL gene IDs were cleaned, and all columns were coerced to numeric format. Low-expression genes were filtered out by retaining those with a mean count $\geq$200 across samples. Gene annotation was performed using *org.Hs.*e.g.,*.db* and mapIds.

Sample metadata were loaded and reordered to match the count matrix. A DESeqDataSet was constructed (design: ~ patient), and normalization was applied using DESeq2 size factors. Quality control included PCA visualization using plotPCA, ggplot2, and ggrepel.

Differential expression analysis was performed with DESeq2, and genes with an adjusted $p < 0.05$ were considered significantly differentially expressed. MA plots and dispersion estimates were generated to assess model fit. Differentially expressed genes were ranked by p value, annotated using AnnotationDbi, and the top 100 differentially expressed genes (DEGs) were exported for downstream analyses and visualization with the pheatmap R package (https://cran.r-project.org/web/packages/pheatmap/index.html).

Statistical analysis

Graphing and analysis were performed using Prism 9.0.1 (GraphPad, California, USA). Significance was assessed using a repeated measures (RM) one-way ANOVA with Greenhouse-Geiser correction and Dunnett's test for multiple comparisons, a mixed-effects analysis with Sidak's multiple comparison test, or a one-tailed paired t test. Data are reported as mean ± standard deviation (SD). A p value below 0.05 was considered statistically significant.

DATA AND CODE AVAILABILITY

Sequencing data has been depostied under BioProject: PRJNA1427148. Other data are available upon request from the corresponding author.

ACKNOWLEDGMENTS

This work was supported by funding from the Natural Sciences and Engineering Research Council of Canada (RGPIN-2019-05042), the "Chaire de Recherche Banque de Montreal (BMO)," and the "Fonds d'innovation thérapeutique (FIT2023-2024)" from the Fondation Charles Bruneau to E.H. M.B. is the recipient of the Fonds de recherche du Québec – Santé (FRQS) doctoral scholarship (370454), the Cole Foundation fellowship, and the Bourse d'excellence en médecine régénératrice from the Université de Montréal. We thank our blood donors for their contribution.

AUTHOR CONTRIBUTIONS

M.B.: Conception of study, experimental design, experimentation, data analysis, and writing and review. A.B.: Experimental design, data analysis, and manuscript review. H.R.: Construct cloning and manuscript review. K.B.: manuscript review. E.G.: Supervision, provision of resources, experimental design, and manuscript review. E.H.: Supervision, provision of resources, experimental design, funding acquisition, and manuscript review.

DECLARATION OF INTERESTS

The authors declare no competing interests.

SUPPLEMENTAL INFORMATION

Supplemental information can be found online at https://doi.org/10.1016/j.omta.2026.201692.

REFERENCES

1. Liu, E., Marin, D., Banerjee, P., Macapinlac, H.A., Thompson, P., Basar, R., Nassif Kerbauy, L., Overman, B., Thall, P., Kaplan, M., et al. (2020). Use of CAR-Transduced Natural Killer Cells in CD19-Positive Lymphoid Tumors. N. Engl. J. Med. *382*, 545–553. https://doi.org/10.1056/NEJMoa1910607.

2. Grupp, S.A., Kalos, M., Barrett, D., Aplenc, R., Porter, D.L., Rheingold, S.R., Teachey, D.T., Chew, A., Hauck, B., Wright, J.F., et al. (2013). Chimeric Antigen Receptor–Modified T Cells for Acute Lymphoid Leukemia. N. Engl. J. Med. *368*, 1509–1518. https://doi.org/10.1056/NEJMoa1215134.

3. Gentner, B., Tucci, F., Galimberti, S., Fumagalli, F., De Pellegrin, M., Silvani, P., Camesasca, C., Pontesilli, S., Darin, S., Ciotti, F., et al. (2021). Hematopoietic Stem- and Progenitor-Cell Gene Therapy for Hurler Syndrome. N. Engl. J. Med. *385*, 1929–1940. https://doi.org/10.1056/NEJMoa2106596.

4. Müller, F., Taubmann, J., Bucci, L., Wilhelm, A., Bergmann, C., Völkl, S., Aigner, M., Rothe, T., Minopoulou, I., Tur, C., et al. (2024). CD19 CAR T-Cell Therapy in Autoimmune Disease — A Case Series with Follow-up. N. Engl. J. Med. *390*, 687–700. https://doi.org/10.1056/NEJMoa2308917.

5. Ayala Ceja, M., Khericha, M., Harris, C.M., Puig-Saus, C., and Chen, Y.Y. (2024). CAR-T cell manufacturing: Major process parameters and next-generation strategies. J. Exp. Med. *221*, e20230903. https://doi.org/10.1084/jem.20230903.

6. Jadlowsky, J.K., Leskowitz, R., McKenna, S., Karar, J., Ma, Y., Dai, A., Plesa, G., Chen, F., Alexander, K., Petrella, J., et al. (2024). Long-term stability of clinical-grade lentiviral vectors for cell therapy. Mol. Ther. Methods Clin. Dev. *32*, 101186. https://doi.org/10.1016/j.omtm.2024.101186.

7. Bari, R., Granzin, M., Tsang, K.S., Roy, A., Krueger, W., Orentas, R., Schneider, D., Pfeifer, R., Moeker, N., Verhoeyen, E., et al. (2019). A Distinct Subset of Highly Proliferative and Lentiviral Vector (LV)-Transducible NK Cells Define a Readily Engineered Subset for Adoptive Cellular Therapy. Front. Immunol. *10*, 2001. https://doi.org/10.3389/fimmu.2019.02001.

8. Colamartino, A.B.L., Lemieux, W., Bifsha, P., Nicoletti, S., Chakravarti, N., Sanz, J., Roméro, H., Selleri, S., Béland, K., Guiot, M., et al. (2019). Efficient and Robust NK-Cell Transduction With Baboon Envelope Pseudotyped Lentivector. Front. Immunol. *10*, 2873. https://doi.org/10.3389/fimmu.2019.02873.

9. Renner, A., Stahringer, A., Ruppel, K.E., Fricke, S., Koehl, U., and Schmiedel, D. (2024). Development of KoRV-pseudotyped lentiviral vectors for efficient gene transfer into freshly isolated immune cells. Gene Ther. *31*, 378–390. https://doi.org/10.1038/s41434-024-00454-0.

10. Kim, Y., Lee, D.Y., Choi, J.U., Park, J.S., Lee, S.M., Kang, C.H., and Park, C.H. (2023). Optimized conditions for gene transduction into primary immune cells using viral vectors. Sci. Rep. *13*, 12365. https://doi.org/10.1038/s41598-023-39597-2.

11. Gong, Y., Klein Wolterink, R.G.J., Janssen, I., Groot, A.J., Bos, G.M.J., and Germeraad, W.T.V. (2020). Rosuvastatin Enhances VSV-G Lentiviral Transduction of NK Cells via Upregulation of the Low-Density Lipoprotein Receptor. Mol. Ther. Methods Clin. Dev. *17*, 634–646. https://doi.org/10.1016/j.omtm.2020.03.017.

12. Chockley, P., Patil, S.L., and Gottschalk, S. (2021). Transient blockade of TBK1/IKKε allows efficient transduction of primary human natural killer cells with vesicular

stomatitis virus G-pseudotyped lentiviral vectors. Cytotherapy *23*, 787–792. https://doi.org/10.1016/j.jcyt.2021.04.010.

13. Allan, D.S.J., Chakraborty, M., Waller, G.C., Hochman, M.J., Poolcharoen, A., Reger, R.N., and Childs, R.W. (2021). Systematic improvements in lentiviral transduction of primary human natural killer cells undergoing ex vivo expansion. Mol. Ther. Methods Clin. Dev. *20*, 559–571. https://doi.org/10.1016/j.omtm.2021.01.008.

14. Ma, J., Zhu, Y., Kong, J., Yu, D., Toh, W.H., Jain, M., Ni, Q., Ge, Z., Lin, J., Choy, J., et al. (2024). Tuning extracellular fluid viscosity to enhance transfection efficiency. Nat. Chem. Eng. *1*, 576–587. https://doi.org/10.1038/s44286-024-00116-3.

15. Noguchi, K., Ikawa, Y., Takenaka, M., Sakai, Y., Fujiki, T., Kuroda, R., Chappell, M., Ghiaccio, V., Rivella, S., and Wada, T. (2023). Protocol for a high titer of BaEV-Rless pseudotyped lentiviral vector: Focus on syncytium formation and detachment. J. Virol. Methods *314*, 114689. https://doi.org/10.1016/j.jviromet.2023.114689.

16. Klatt, D., Sereni, L., Liu, B., Genovese, P., Schambach, A., Verhoeyen, E., Williams, D.A., and Brendel, C. (2024). Engineered packaging cell line for the enhanced production of baboon-enveloped retroviral vectors. Mol. Ther. Nucleic Acids *35*, 102389. https://doi.org/10.1016/j.omtn.2024.102389.

17. Rennick, J.J., Johnston, A.P.R., and Parton, R.G. (2021). Key principles and methods for studying the endocytosis of biological and nanoparticle therapeutics. Nat. Nanotechnol. *16*, 266–276. https://doi.org/10.1038/s41565-021-00858-8.

18. Sun, X., Yau, V.K., Briggs, B.J., and Whittaker, G.R. (2005). Role of clathrin-mediated endocytosis during vesicular stomatitis virus entry into host cells. Virology *338*, 53–60. https://doi.org/10.1016/j.virol.2005.05.006.

19. Haso, W., Lee, D.W., Shah, N.N., Stetler-Stevenson, M., Yuan, C.M., Pastan, I.H., Dimitrov, D.S., Morgan, R.A., FitzGerald, D.J., Barrett, D.M., et al. (2013). Anti-CD22-chimeric antigen receptors targeting B-cell precursor acute lymphoblastic leukemia. Blood *121*, 1165–1174. https://doi.org/10.1182/blood-2012-06-438002.

20. Sweeney, N.P., and Vink, C.A. (2021). The impact of lentiviral vector genome size and producer cell genomic to gag-pol mRNA ratios on packaging efficiency and titre. Mol. Ther. Methods Clin. Dev. *21*, 574–584. https://doi.org/10.1016/j.omtm.2021.04.007.

21. Wang, W., Liu, Y., He, Z., Li, L., Liu, S., Jiang, M., Zhao, B., Deng, M., Wang, W., Mi, X., et al. (2024). Breakthrough of solid tumor treatment: CAR-NK immunotherapy. Cell Death Discov. *10*, 40. https://doi.org/10.1038/s41420-024-01815-9.

22. Li, J., Xu, W., Luo, S., Zhang, H., Qiu, X., Zhang, H., Liu, Z., and Pang, Q. (2025). Improving the production of BaEV lentivirus by comprehensive optimization. J. Virol. Methods *333*, 115106. https://doi.org/10.1016/j.jviromet.2024.115106.

23. Bera, K., Kiepas, A., Godet, I., Li, Y., Mehta, P., Ifemembi, B., Paul, C.D., Sen, A., Serra, S.A., Stoletov, K., et al. (2022). Extracellular fluid viscosity enhances cell migration and cancer dissemination. Nature *611*, 365–373. https://doi.org/10.1038/s41586-022-05394-6.

24. Kieser, Q.J., Granoski, M.J., McClelland, R.D., Griffiths, C., Bilawchuk, L.M., Stojic, A., Elawar, F., Jamieson, K., Proud, D., and Marchant, D.J. (2023). Actin cytoskeleton remodeling disrupts physical barriers to infection and presents entry receptors to respiratory syncytial virus. J. Gen. Virol. *104*, 001923. https://doi.org/10.1099/jgv.0.001923.

25. Lim, J.P., and Gleeson, P.A. (2011). Macropinocytosis: an endocytic pathway for internalising large gulps. Immunol. Cell Biol. *89*, 836–843. https://doi.org/10.1038/icb.2011.20.

26. Kruth, H.S. (2011). Receptor-independent fluid-phase pinocytosis mechanisms for induction of foam cell formation with native LDL particles. Curr. Opin. Lipidol. *22*, 386–393. https://doi.org/10.1097/MOL.0b013e32834adadb.

27. Li, Y.-X., Wang, N., Hasan, M.M., and Pang, H.-B. (2022). Co-administration of Transportan Peptide Enhances the Cellular Entry of Liposomes in the Bystander Manner Both In Vitro and In Vivo. Mol. Pharm. *19*, 4123–4134. https://doi.org/10.1021/acs.molpharmaceut.2c00537.

28. Nanbakhsh, A., Best, B., Riese, M., Rao, S., Wang, L., Medin, J., Thakar, M.S., and Malarkannan, S. (2018). Dextran Enhances the Lentiviral Transduction Efficiency of Murine and Human Primary NK Cells. J. Vis. Exp. *55063*, 55063. https://doi.org/10.3791/55063.

29. Gold, G.T., Varma, D.M., Harbottle, D., Gupta, M.S., Stalling, S.S., Taub, P.J., and Nicoll, S.B. (2014). Injectable redox-polymerized methylcellulose hydrogels as potential soft tissue filler materials. J. Biomed. Mater. Res. *102*, 4536–4544. https://doi.org/10.1002/jbm.a.35132.

30. Gardner, R.A., White, C., Elsallab, M., Farnia, S., Fraint, E., Grilley, B., Bateman-House, A., Grupp, S.A., Kenderian, S., Locke, F.L., et al. (2024). ACT To Sustain: Adoptive Cell Therapy To Sustain Access to Non-Commercialized Genetically Modified Cell Therapies. Transplant. Cell. Ther. *30*, 776–787. https://doi.org/10.1016/j.jtct.2024.05.010.

31. Marin, D., Li, Y., Basar, R., Rafei, H., Daher, M., Dou, J., Mohanty, V., Dede, M., Nieto, Y., Uprety, N., et al. (2024). Safety, efficacy and determinants of response of allogeneic CD19-specific CAR-NK cells in CD19+ B cell tumors: a phase 1/2 trial. Nat. Med. *30*, 772–784. https://doi.org/10.1038/s41591-023-02785-8.

32. Somanchi, S.S., and Lee, D.A. (2016). Ex Vivo Expansion of Human NK Cells Using K562 Engineered to Express Membrane Bound IL21. In Natural Killer Cells: Methods and Protocols, S.S. Somanchi, ed. (Springer), pp. 175–193. https://doi.org/10.1007/978-1-4939-3684-7_15.

33. Zhang, X.-Y., La Russa, V.F., Bao, L., Kolls, J., Schwarzenberger, P., and Reiser, J. (2002). Lentiviral vectors for sustained transgene expression in human bone marrow-derived stromal cells. Mol. Ther. *5*, 555–565. https://doi.org/10.1006/mthe.2002.0585.

34. Gao, X.D., Tu, L.-C., Mir, A., Rodriguez, T., Ding, Y., Leszyk, J., Dekker, J., Shaffer, S.A., Zhu, L.J., Wolfe, S.A., and Sontheimer, E.J. (2018). C-BERST: defining subnuclear proteomic landscapes at genomic elements with dCas9-APEX2. Nat. Methods *15*, 433–436. https://doi.org/10.1038/s41592-018-0006-2.

35. Zhang, X.-Y., La Russa, V.F., and Reiser, J. (2004). Transduction of Bone-Marrow-Derived Mesenchymal Stem Cells by Using Lentivirus Vectors Pseudotyped with Modified RD114 Envelope Glycoproteins. J. Virol. *78*, 1219–1229. https://doi.org/10.1128/JVI.78.3.1219-1229.2004.

36. Naeimi Kararoudi, M., Likhite, S., Elmas, E., Yamamoto, K., Schwartz, M., Sorathia, K., De Souza Fernandes Pereira, M., Sezgin, Y., Devine, R.D., Lyberger, J.M., et al. (2022). Optimization and validation of CAR transduction into human primary NK cells using CRISPR and AAV. Cell Rep. Methods *2*, 100236. https://doi.org/10.1016/j.crmeth.2022.100236.

OMTA, Volume *34*

Supplemental information

Modulating viscosity improves lentiviral transduction of NK cells: A simple solution to a persistent problem

Mila Bjelica, Aissa Benyoucef, Hugo Romero, Kathie Beland, Etienne Gagnon, and Elie Haddad

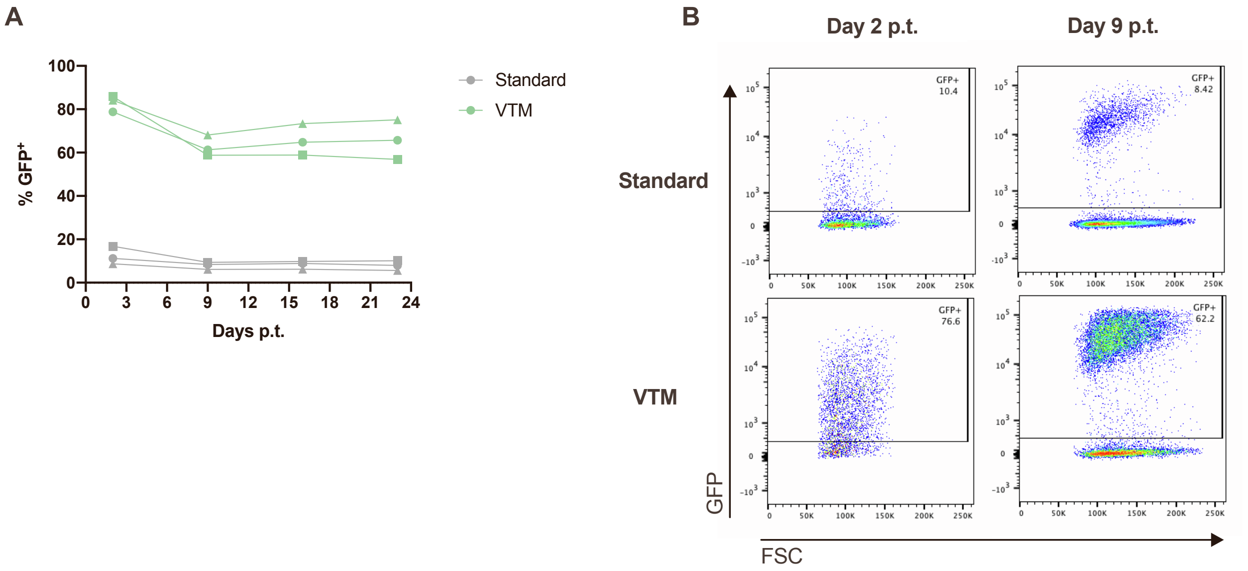

Figure S1. GFP over time A) GFP expression in NK cells post transduction (p.t.) with BaEV lentivirus, as NK are expanded and kept in culture. B) Representative flow cytometry plots at Day 2 and Day 9 post transduction. *n*=3 biological replicates.

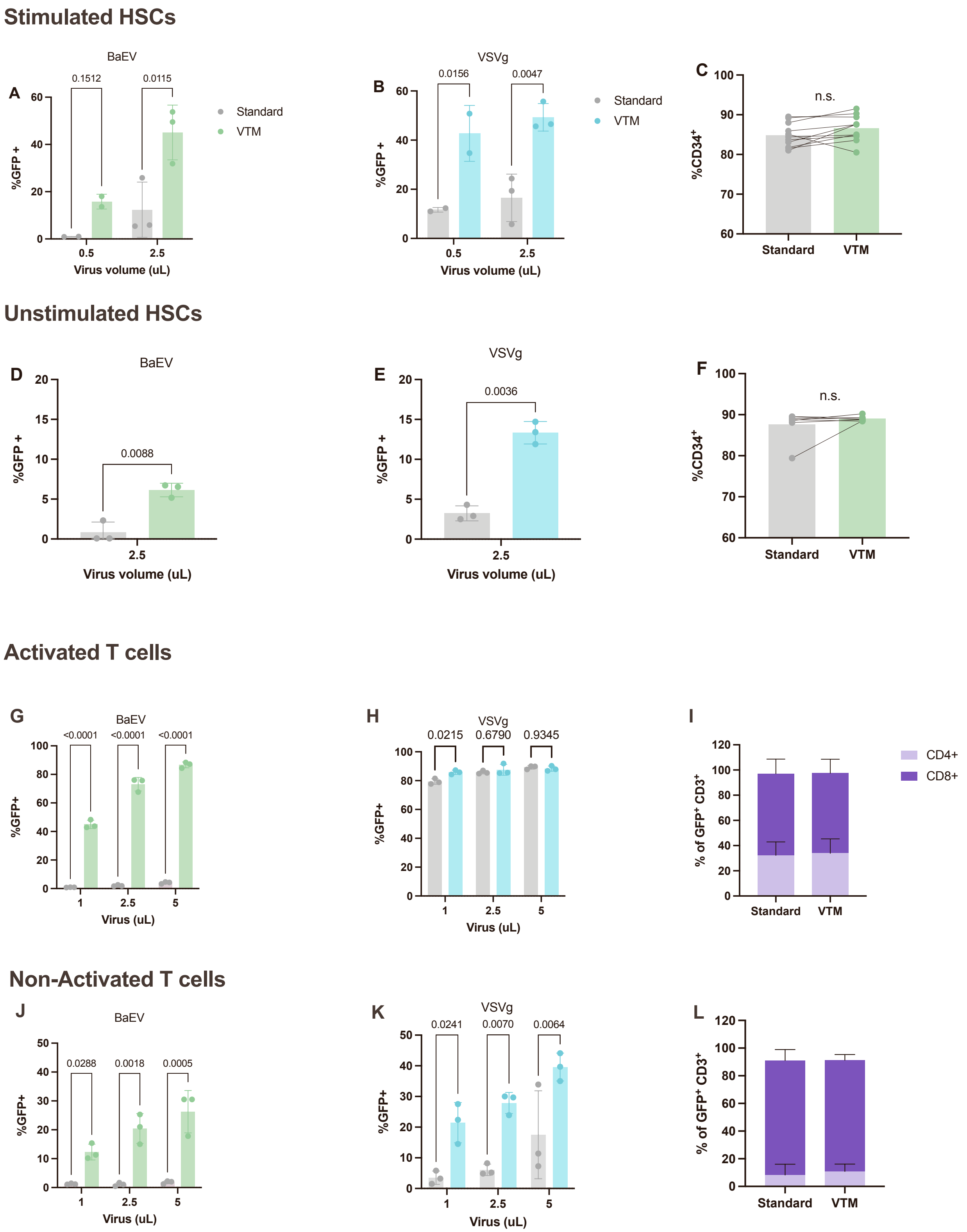

Figure S2. Transduction of HSCs and T cells in standard medium or VTM. (A-C) HSC were put in expansion medium overnight as stimulation prior to transduction, then expanded for 6 more days. A) Transduction efficiency HSCs with BaEV lentivirus at day

6 post transduction. B) Transduction efficiency of HSC with VSVg lentivirus at day 6 post transduction. C) %CD34+ of all samples pooled. (D-F) HSC were isolated and directly transduced then put in expansion for 6 days. D) Transduction efficiency of unstimulated HSC with BaEV lentivirus at day 6 post transduction. E) Transduction efficiency of unstimulated HSC with VSVg lentivirus at day 6 post transduction. F) %CD34+ of all samples pooled (G-I) T cells isolated and activated overnight prior to transduction, then expanded for 3 days. G) Transduction efficiency of T cells with BaEV lentivirus at day 3 post transduction. H) Transduction efficiency of T cells with VSVg lentivirus at day 3 post transduction. I) CD4 and CD8 populations in GFP+ CD3+ T cells all pooled. (J-L) T cells isolated and rested in RPMI for 3 hours prior to transduction, then kept in culture for 3 days. J) Transduction efficiency of T cells with BaEV lentivirus at day 3 post transduction. K) Transduction efficiency of T cells with VSVg lentivirus at day 3 post transduction. L) CD4 and CD8 populations in GFP+ CD3+ T cells all pooled. Significance was determined using a mixed-effects analysis with Sidak's multiple comparison test with a single pooled variance for experiments with multiple viral volumes or a paired single tail t test for experiments with a single viral volume. P values are reported above each comparison.

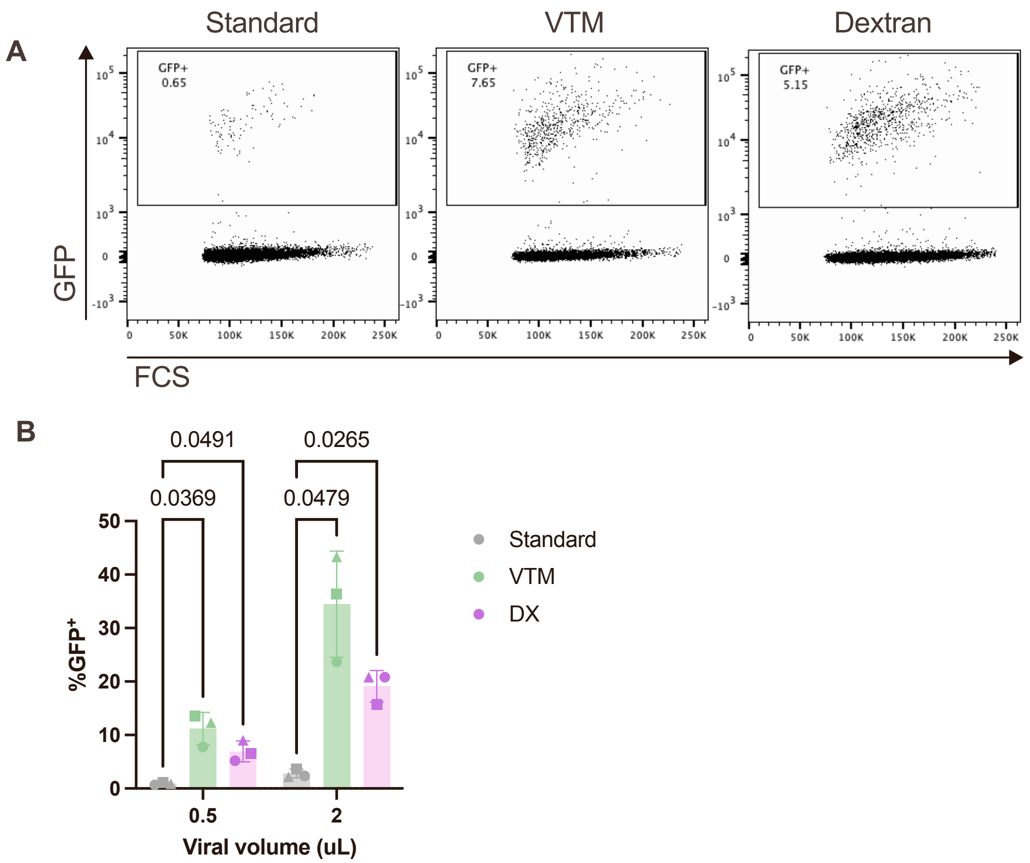

Figure S3. Dextran as an alternative thickening agent. A) Representative flow cytometry plots at Day 9 post transduction. B) Transduction efficiency of NK using BaEV lentivirus and standard medium, VTM or 3cps dextran (DX) supplemented medium at Day 9 post transduction. *n*=3 biological replicates. P values are reported above each comparison.

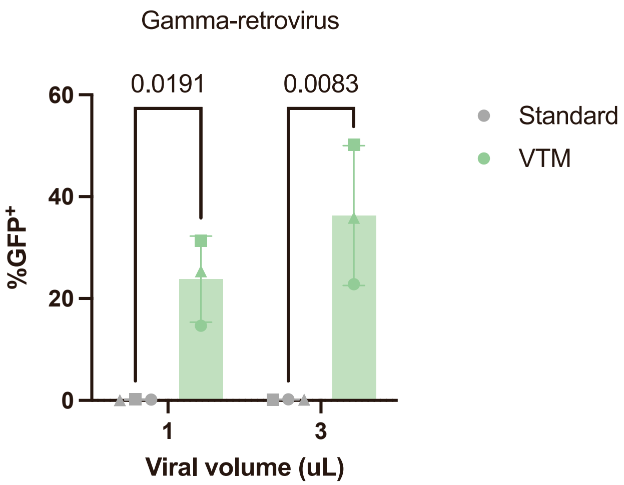

Figure S4. Transduction of NK cells with gamma-retrovirus in standard medium or VTM. A) Transduction efficiency with gamma-retrovirus at day 9 post transduction. *n*=3 biological replicates. P values are reported above each comparison.

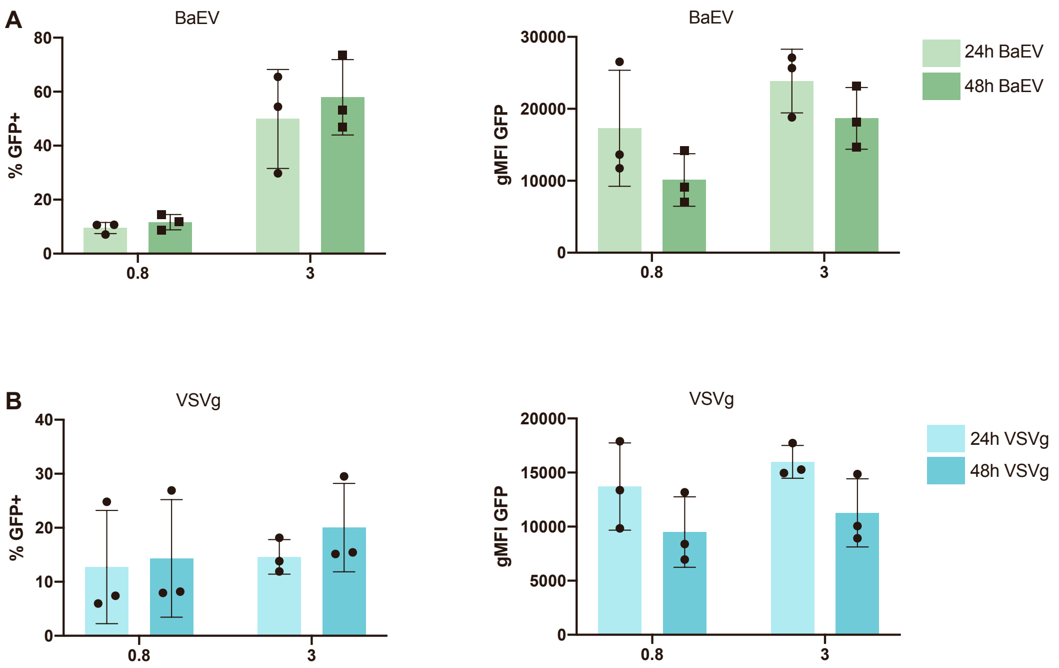

Figure S5. Transduction of NK using a 24 or 48 hour incubation with virus. A) Transduction efficiency with BaEV pseudotyped lentivirus at day 9 post transduction. B) Transduction efficiency with VSVg pseudotyped lentivirus at day 9 post transduction. *n*=3 biological replicates.

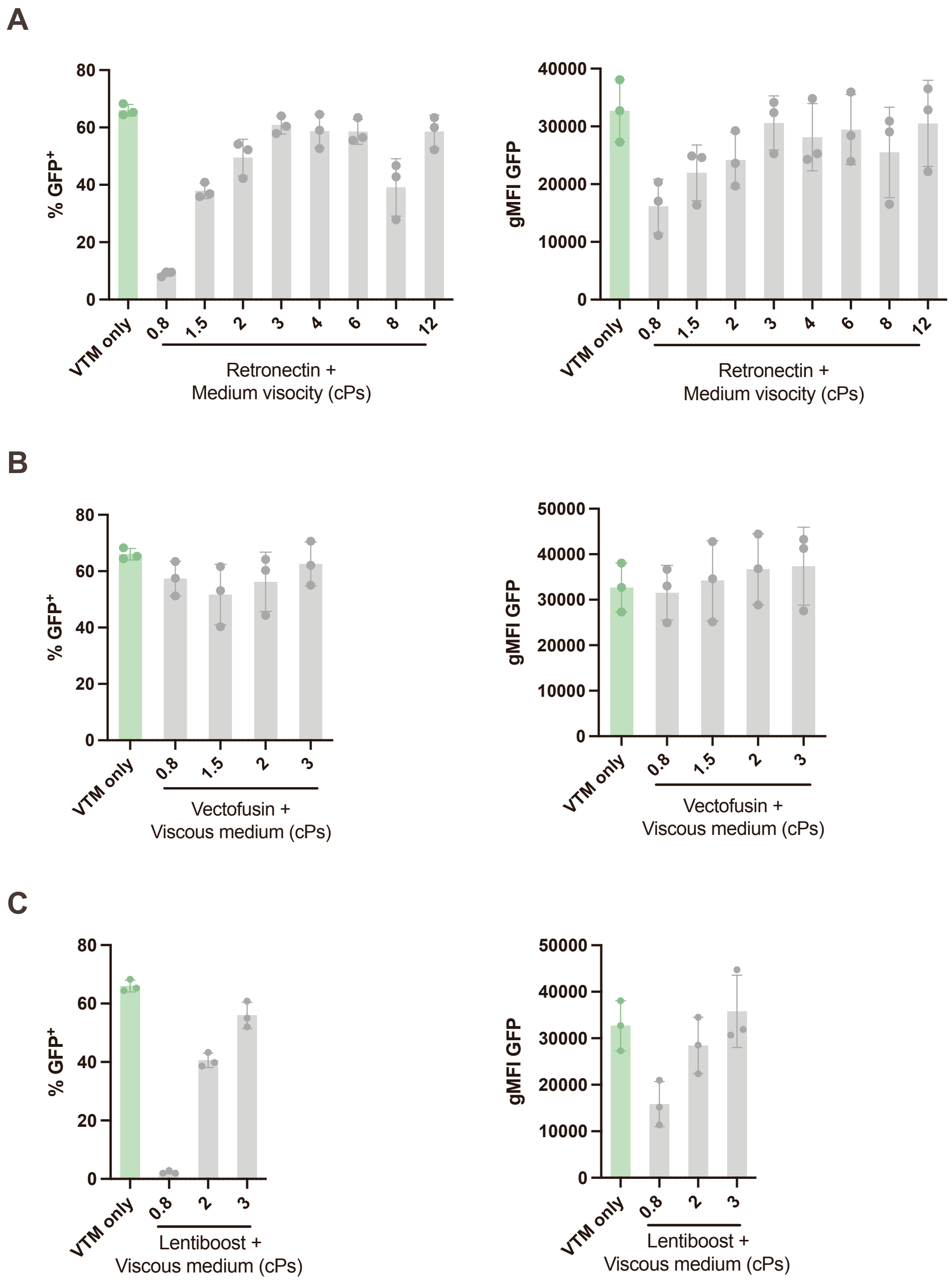

Figure S6. Additive effects of combining commercial transduction enhancers with viscous medium. Transduction efficiency and MFI of NK cells transduced with BaEV GFP in only 3cPs VTM or VTM of varying viscosity in combination with A) Retronectin B) Vectofusin C) Lentiboost. *n*=3 biological replicates. P values are reported above each comparison.

Table S1. Medium viscosity with corresponding methyl-cellulose concentrations. Adapted from Ma et al.[14]

**Medium viscosity (cPs)**	**Methyl-cellulose (w/v%)**
1.0	0.110
1.5	0.210
2.0	0.281
3.0	0.382
4.0	0.453
6.0	0.554
8.0	0.625
12.0	0.725
15.0	0.781
